# Supplementary material for: Human expansion into Asian highlands in the 21st Century and its effects
Source: Nat Commun. 2022 Aug 24;13:4955. doi: 10.1038/s41467-022-32648-8 (PMC9402921; doi:10.1038/s41467-022-32648-8)
Supplement: Supplementary file 1 — Supplementary Information [file 41467_2022_32648_MOESM1_ESM.pdf]

Supplementary Information for

Human expansion into Asian highlands in the 21st Century and its effects

Chao Yang [1, 2], Huizeng Liu [1, 3], Qingquan Li [1 *], Xuqing Wang [4], Wei Ma [5], Cuiling Liu [1, 2], Xu Fang [1, 6], Yuzhi Tang[1], Tiezhu Shi[1, 2], Qibiao Wang [7], Yue Xu[1], Jie Zhang[8], Xuecao Li [9], Gang Xu [10], Junyi Chen[11], Mo Su[12], Shuying Wang [1], Jinjing Wu[1], Leping Huang [1], Xue Li [1], Guofeng Wu [1, 2 *]

1. MNR Key Laboratory for Geo-Environmental Monitoring of Great Bay Area & Guangdong-Hong Kong-Macau Joint Laboratory for Smart Cities & Guangdong Key Laboratory of Urban Informatics & Shenzhen Key Laboratory of Spatial Smart Sensing and Services, Shenzhen University, Shenzhen 518060, China;
2. School of Architecture and Urban Planning, Shenzhen University, Shenzhen 518060, China;
3. Institute for Advanced Study, Shenzhen University, Shenzhen 518060, China;
4. Center for Hydrogeology and Environmental Geology, China Geological Survey, Nanjing 210000, China;
5. School of Civil Engineering, Chongqing Jiaotong University, Chongqing 400074, China;
6. College of Electronics and Information engineering, Shenzhen University, Shenzhen 518060, China;
7. Anhui Zhonghui Urban Planning Survey & Design Institute, Tongling 244000, China;
8. College of Information and Electrical engineering, China Agricultural University, Beijing 100083, China;
9. College of Land Science and Technology, China Agricultural University, Beijing 100083, China;
10. School of Resource and Environmental Sciences, Wuhan University, Wuhan 430079, China;
11. Key Laboratory of Virtual Geographic Environment of the Ministry of Education, Nanjing Normal University, Nanjing 210000, China;
12. Shenzhen Urban Planning and Land Resource Research Center, Shenzhen 518034, China

This PDF file includes:

Supplementary Figures 1 to 13

Supplementary Tables 1 to 10

Supplementary references

Supplementary Figure 1. Typical human activity expansion in highland (artificial surface expansion in East Asia, 25°39' 45.35"N, 100°19' 26.93"E). The images were obtained from Google Earth Pro®.

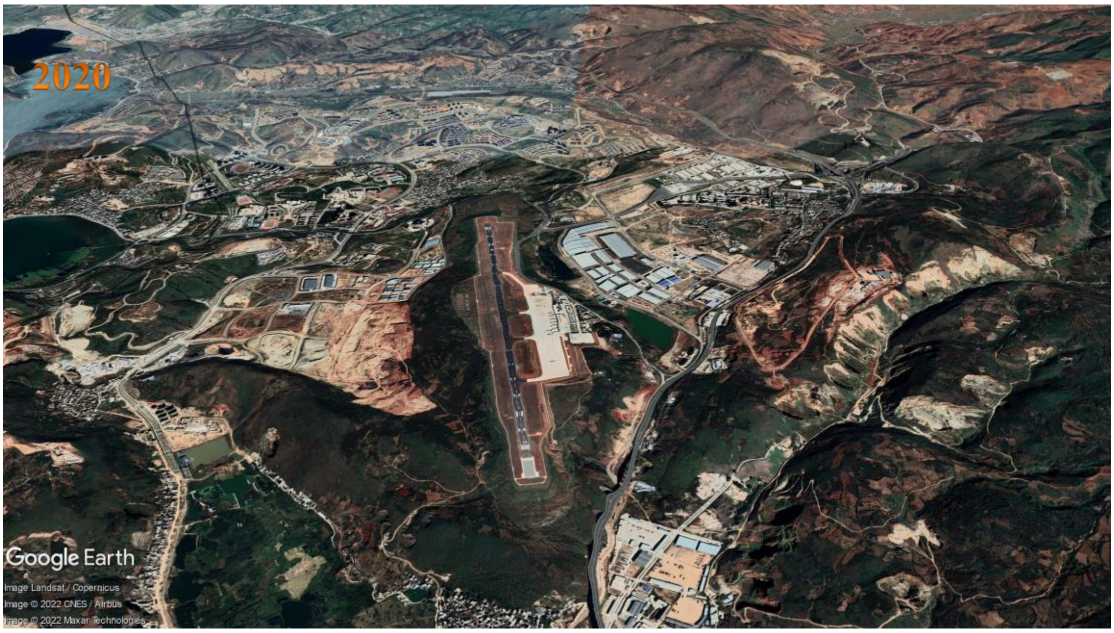

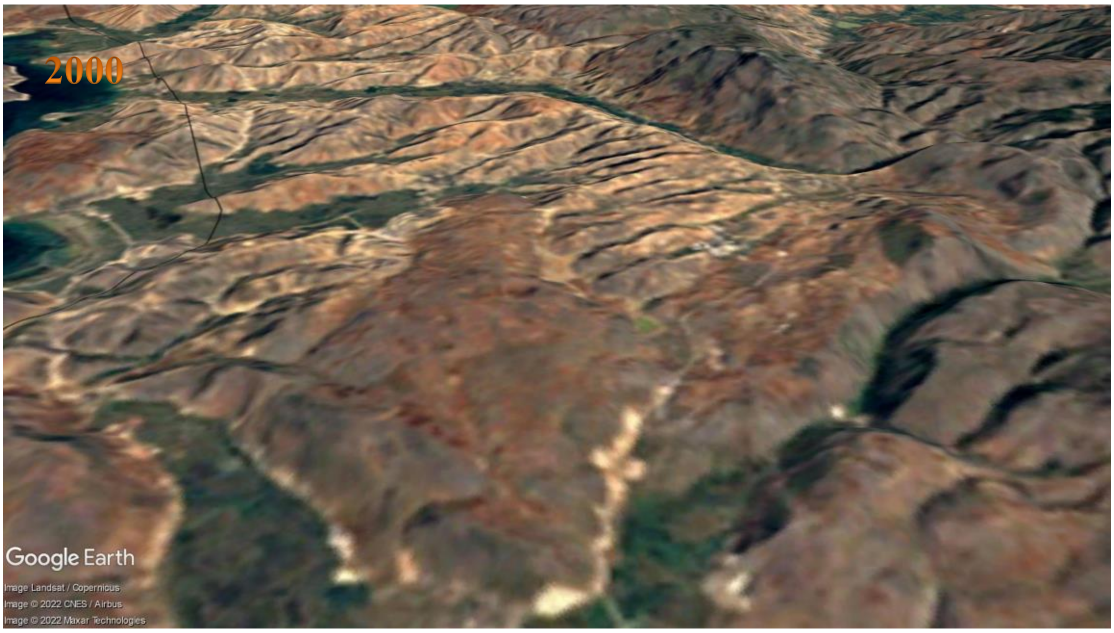

Supplementary Figure 2. Typical human activity expansion in highland (cultivated land expansion in Southeast Asia, 12°00'41.97"N, 108°28'18.02"E). The images were obtained from Google Earth Pro®.

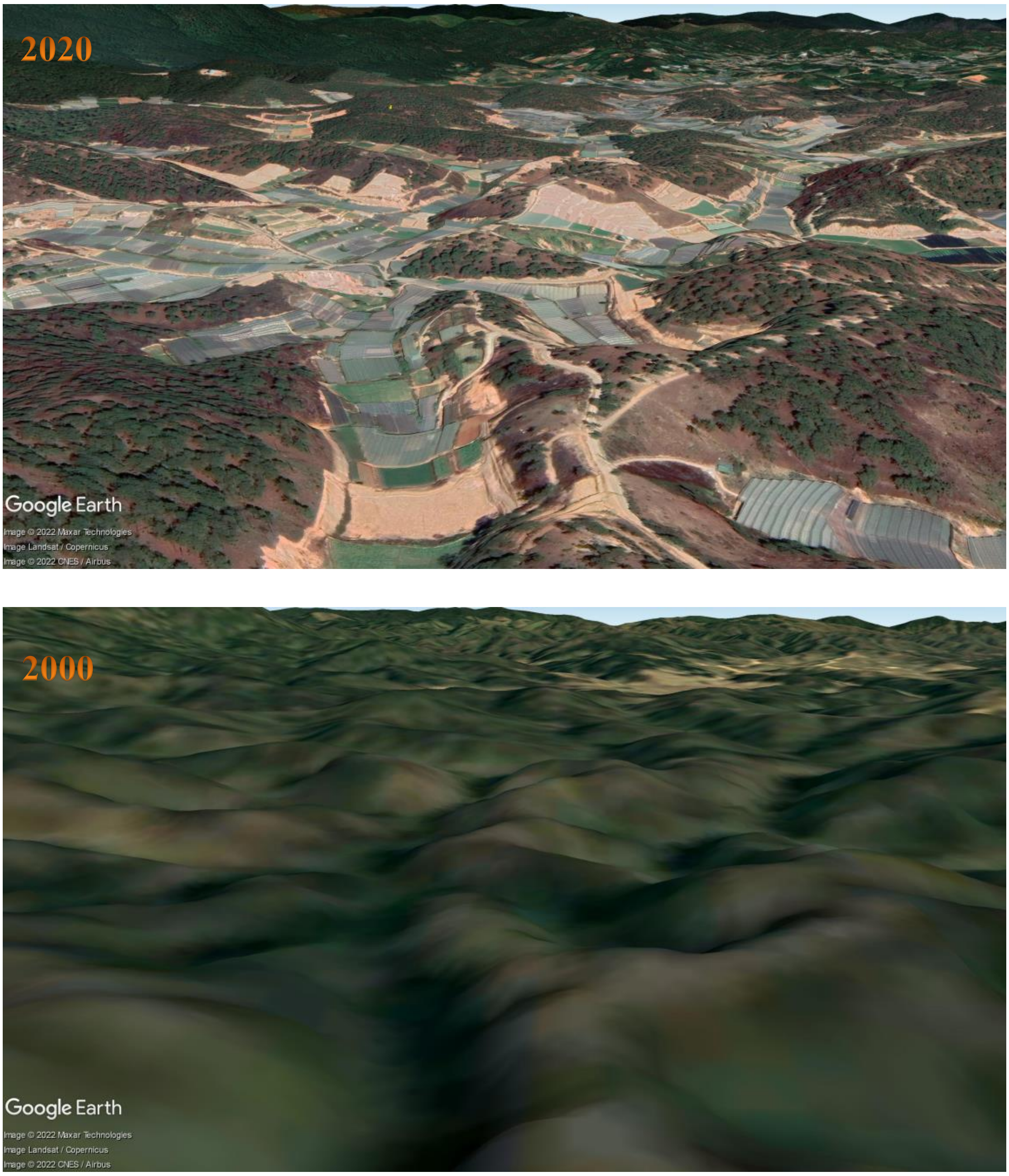

Supplementary Figure 3. Typical human activity expansion in highland (artificial surface expansion in South Asia, 18°47'13.28"N, 78°16'49.22"E). The images were obtained from Google Earth Pro®.

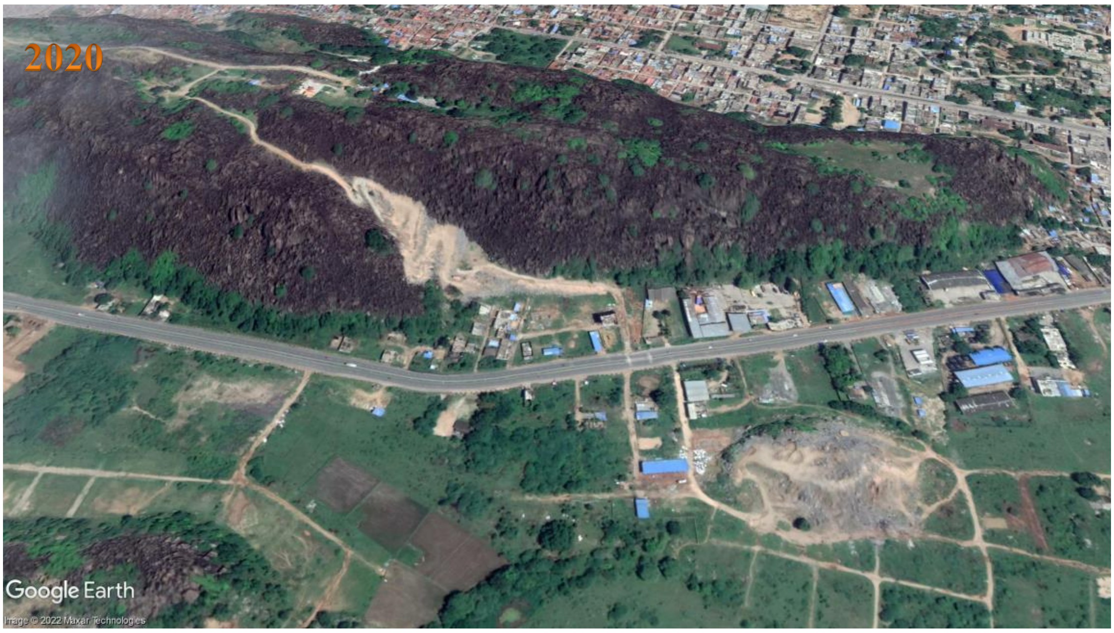

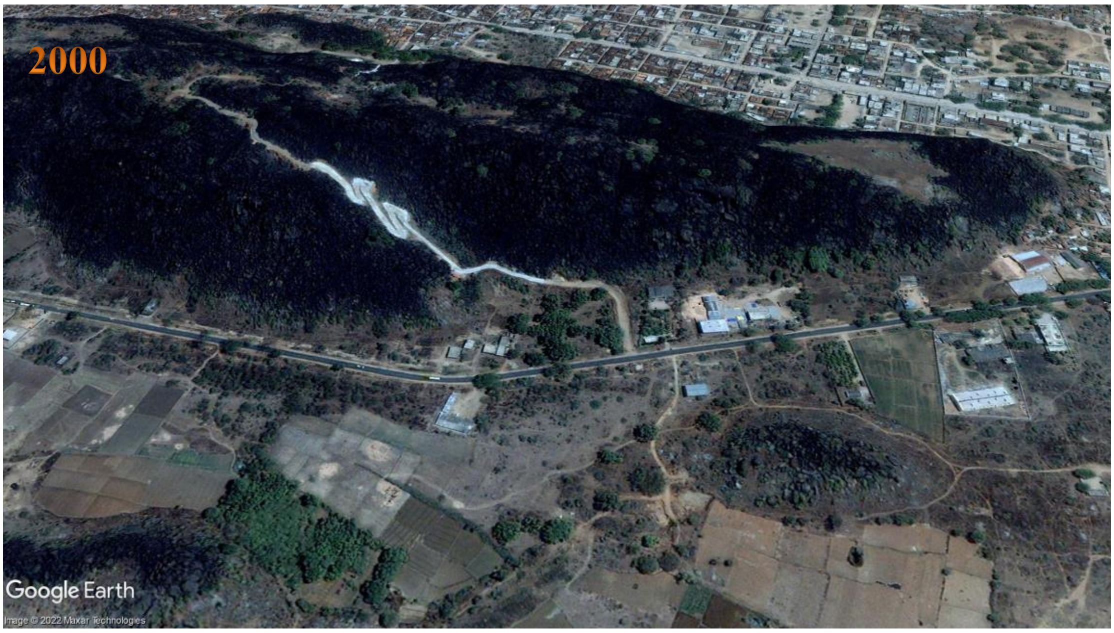

Supplementary Figure 4. Typical human activity expansion in highland (artificial surface expansion in West Asia, 37°06'38.47"N, 35°28'05.09"E). The images were obtained from Google Earth Pro®.

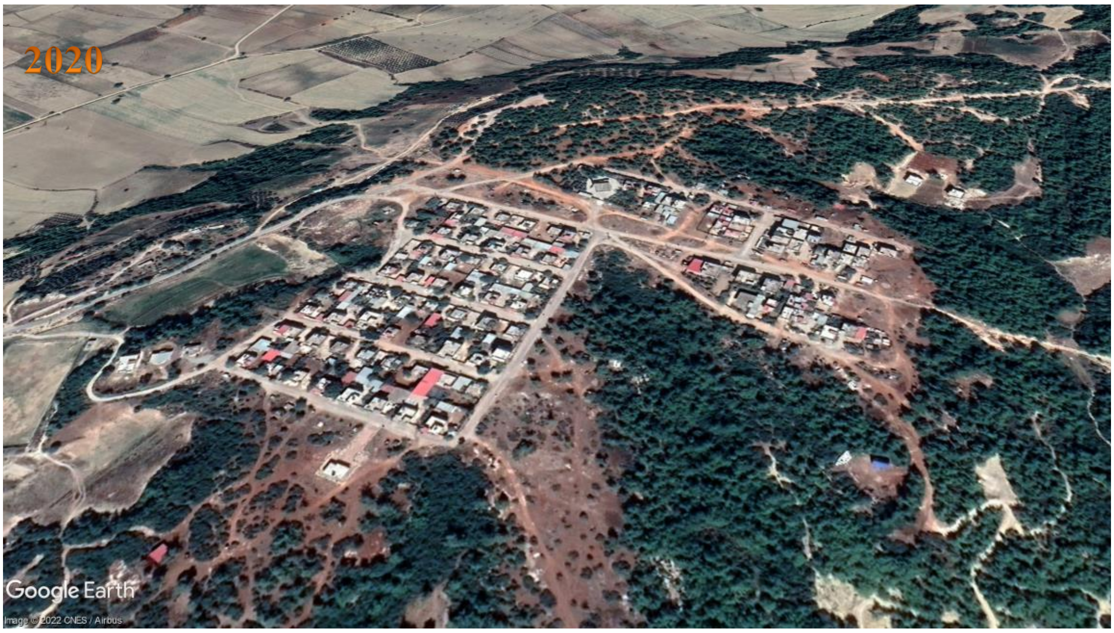

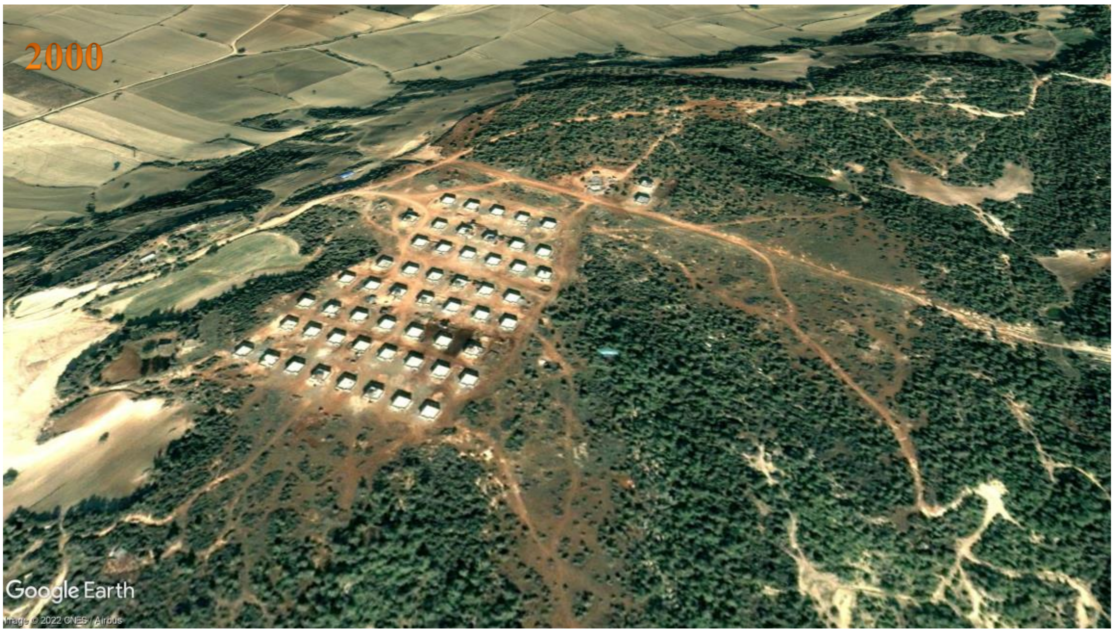

Supplementary Figure 5. Typical human activity expansion in highland (artificial surface expansion in Central Asia, 55°13'22.82"N, 61°25'43.49"E). The images were obtained from Google Earth Pro®.

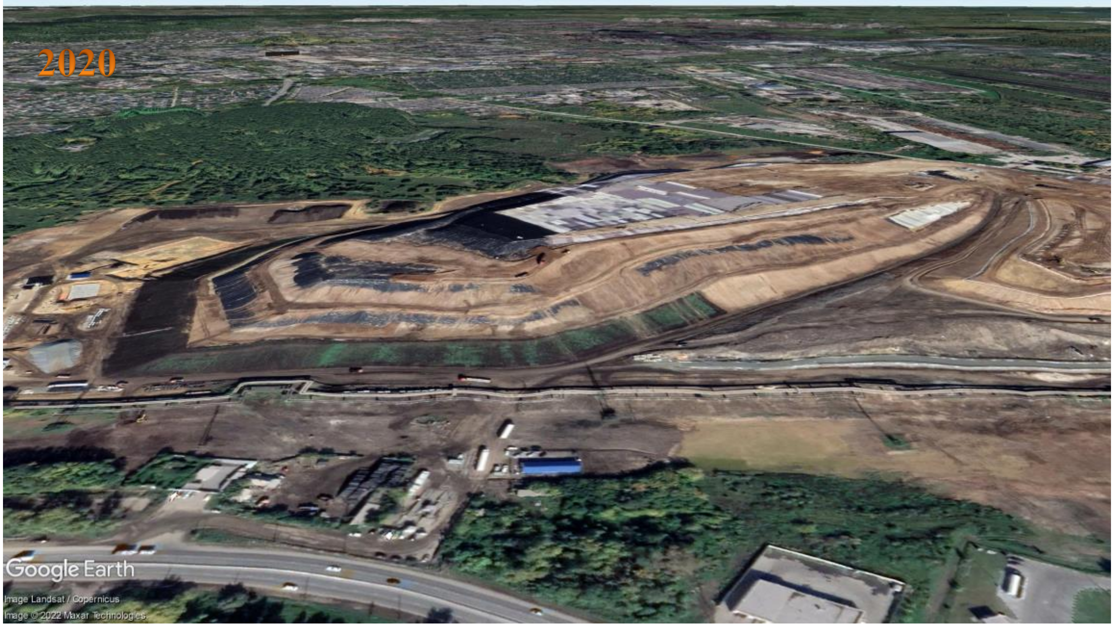

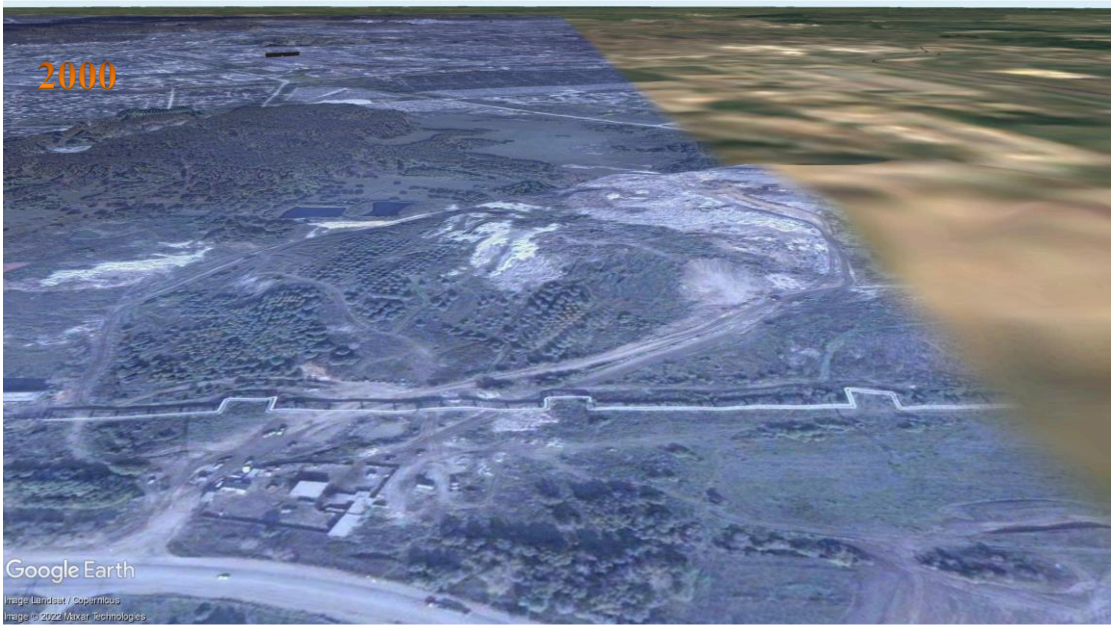

Supplementary Figure 6. Distribution of samples for validating human activity expansion.

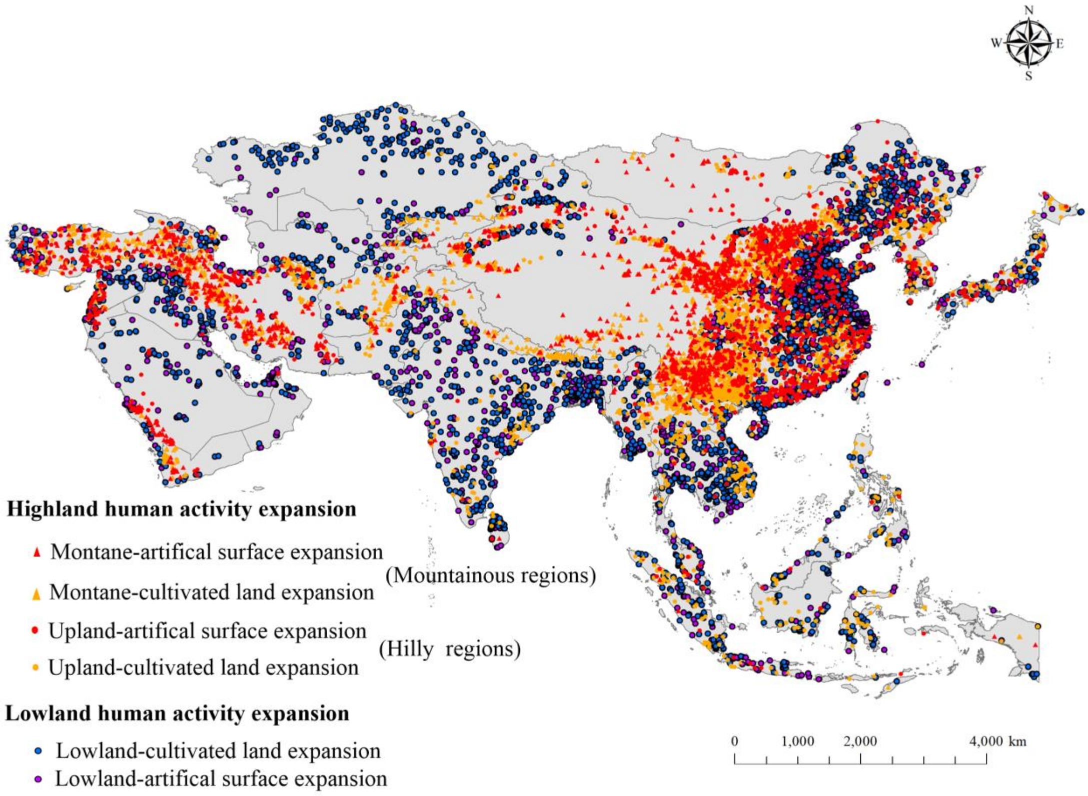

Supplementary Figure 7. Distribution of samples for validating ecological land loss induced by human activity expansion.

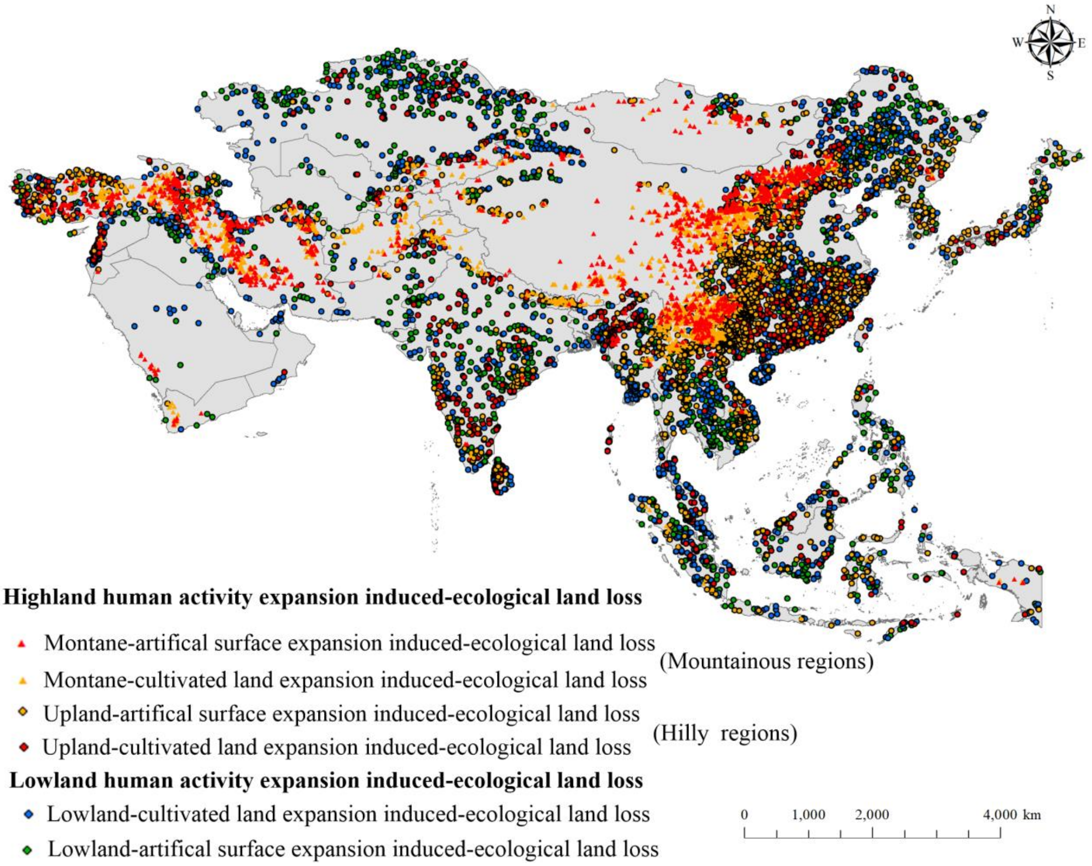

Supplementary Figure 8. Human activity expansion rate in highland at country level. (a) Rank of artificial surface expansion rate in highland; **(b)** Rank of cultivated land expansion rate in highland.

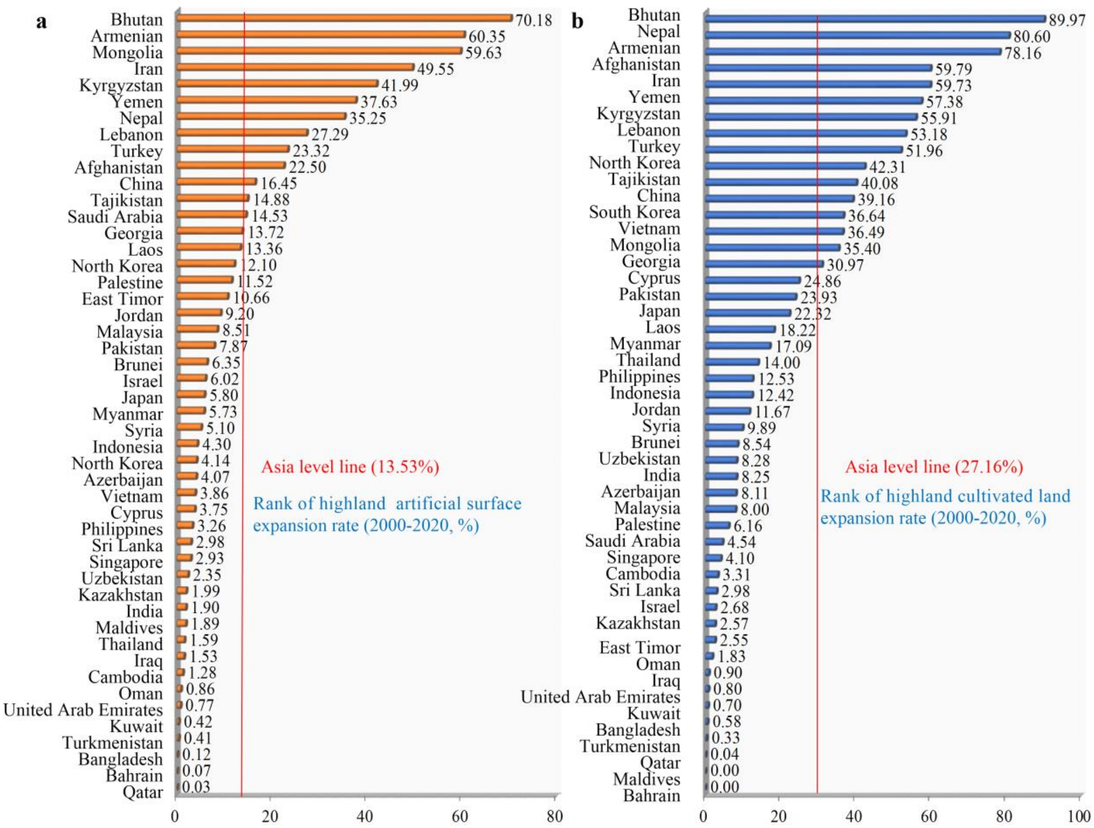

Supplementary Figure 9. Ecological land loss rate in highland induced by human activity expansion and the proportion of loss types in 48 Asian countries. (a) Rank of ecological land loss rate**; (b)** the proportion of ecological land loss type.

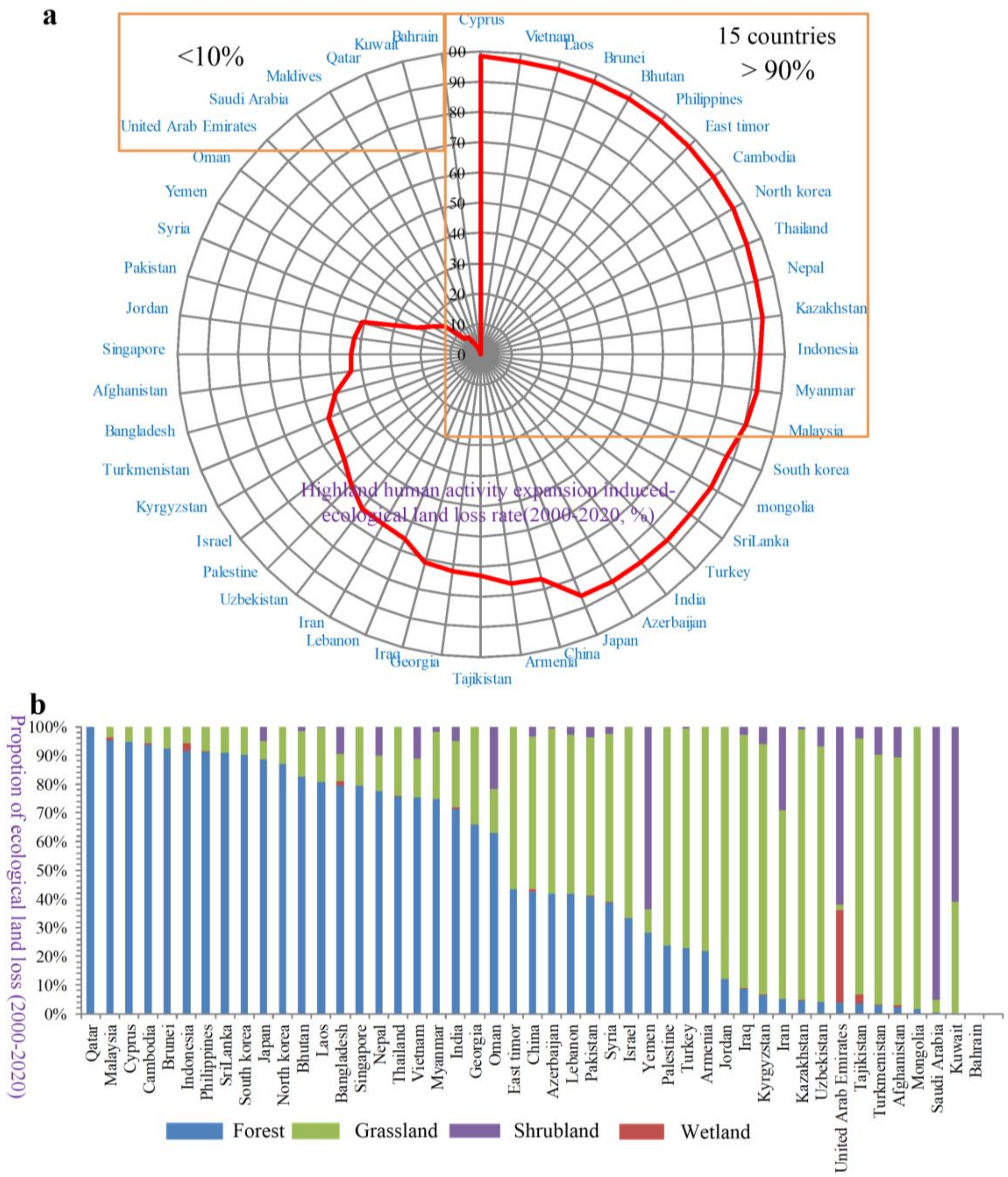

Supplementary Figure 10. Value of habitat fragmentation index (HFI) of ecological land in highland in Asia. (a) Trend of HFI value in Asian hills in 2000-2020; **(b)** Trend of HFI value in Asian mountains in 2000-2020.

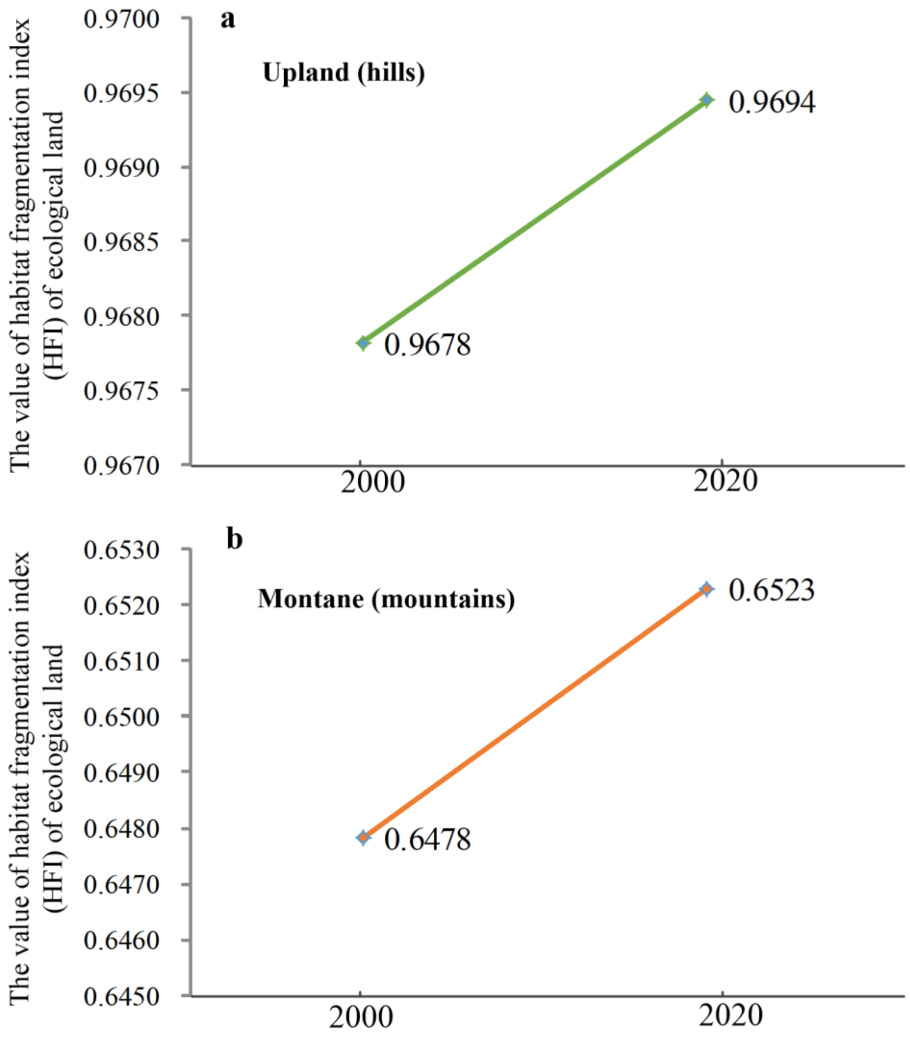

Supplementary Figure 11. Ecological land loss rate in highland induced by different human activity expansion types in 48 Asian countries. (a) Rank of ecological land loss rate in highland induced by cultivated expansion; **(b)** Rank of ecological land loss rate in highland induced by artificial surface expansion.

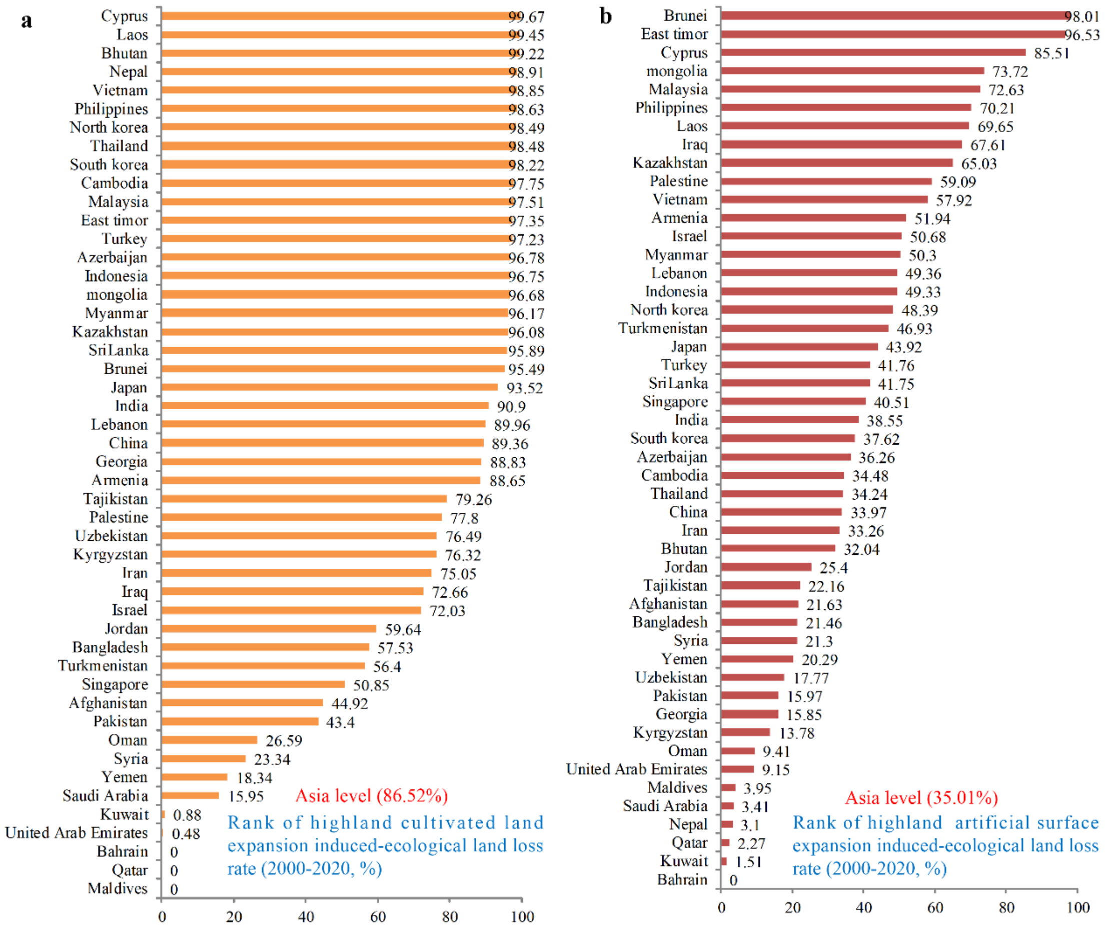

Supplementary Figure 12. Correlation between topographic constraint and highland human activity expansion rate. (a) Highland proportion in 48 Asian countries**; (b)** Correlation between very strong topographic constraint (highland proportion > 95% or < 5%) and human activity expansion rate in highland; **(c)** Correlation between relative strong topographic constraint (highland proportion between 5% - 95%) and human activity expansion rate in highland.

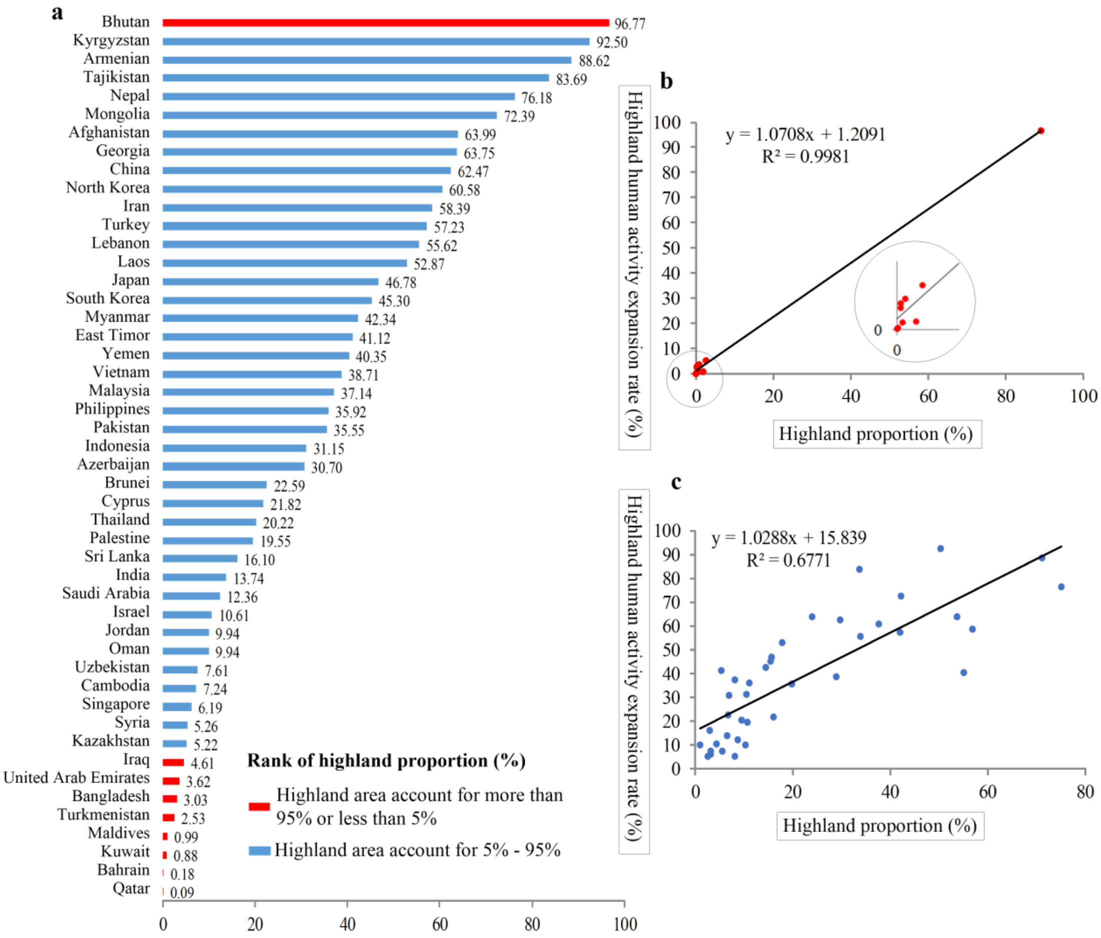

Supplementary Figure 13. Significance test of proportion of human activity expansion types in highlands to economic level. (a) Artificial surface expansion type, Wilcoxon test p<0.01); **(b)** Cultivated land expansion type, Wilcoxon test p<0.01**).**

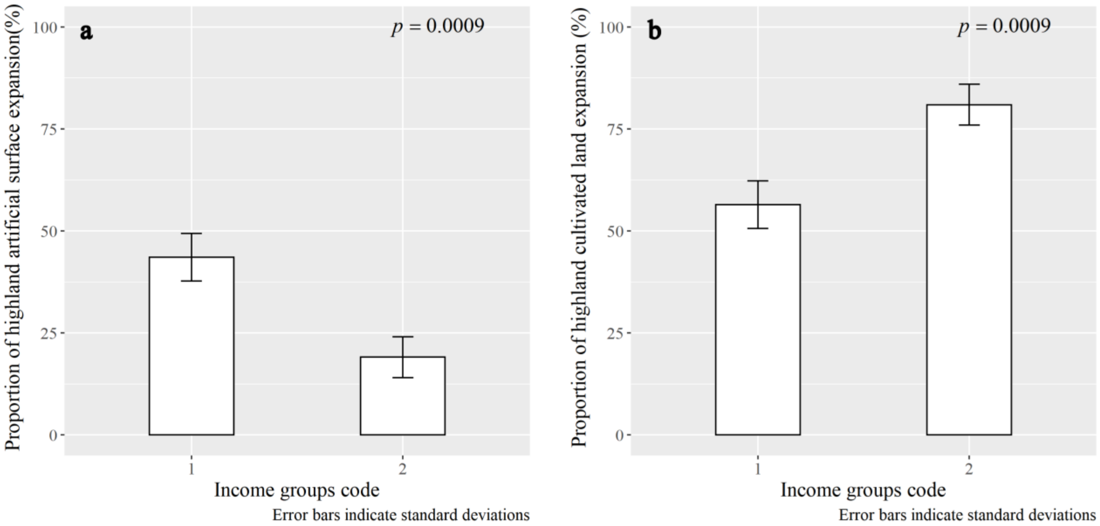

1 High-income or upper-middle-income countries 2 Low-income or lower-middle-income countries

Supplementary Table 1. Accuracy of human activity expansion detection in Asia. HAE is human activity expansion. OA is overall accuracy (i.e., the percent of correct detection). Only the sample with cultivated land or artificial surface in 2020 but not in 2000 was counted as HAE.

2000-2020	**Highland HAE**				
	Upland (hills)		Montane (mountains)		
Samples counts	upland-cultivated land expansion	upland-artificial surface expansion	montane-artificial surface expansion	montane-cultivate d land expansion	Total
Selected samples	1732	1688	1678	1578	6676
Valid samples	1716	1668	1665	1578	6627
Correct samples	1578	1565	1647	1382	6172
OA (%)	**93.13%**				
	**Lowland HAE**				
Samples counts	lowland-cultivated land expansion		lowland-artificial surface expansion		Total
Selected samples	2059		1949		4008
Valid samples	2059		1942		4001
Correct samples	2036		1757		3793
OA (%)	**94.80%**				

Supplementary Table 2. Accuracy of ecological land loss detection induced by human activity expansion in Asia. HELL is ecological land loss induced by human activity expansion. OA is overall accuracy (i.e., the percent of correct detection). Only the sample with ecological land (i.e., forest, grassland, wetland or shrub) in 2000 but with cultivated land or artificial surface in 2020 was counted as HELL.

2000-2020	**Highland HELL**				
	Upland (hills)		Montane (mountains)		
Samples counts	upland-cultivated land expansion induced-ecological land loss	upland-artificial surface expansion induced- ecological land loss	montane-artificial surface expansion induced ecological-land loss	montane-cultivated land expansion induced-ecological land loss	Total
Selected samples	1503	1666	1604	1502	6275
Valid samples	1500	1666	1142	1496	5804
Correct samples	1394	1399	1021	1347	5161
OA (%)	**88.92%**				

	**Lowland HELL**		
Samples counts	lowland-cultivated land expansion induced-ecological land loss	lowland-artificial surface expansion induced-ecological land loss	Total
Selected samples	2142	2332	4474
Valid samples	2142	2325	4467
Correct samples	1896	2055	3951
OA (%)	**88.45%**		

Supplementary Table 3. Statistics of human activity expansion in highlands.

2000-2020	Highland types	Proportion	The proportion of highland cultivated land expansion	Proportion	The proportion of highland artificial surface expansion	Proportion	Highland human activity expansion rate
**Asia**	Upland	47.29	79.75	36.82	20.25	**45.17**	**22.56**
	Montane	52.71		63.18		**54.83**	
Central Asia	Upland	35.94	82.38	31.24	17.62	35.11	**4.45**
	Montane	64.06		68.76		64.89	
South Asia	Upland	47.41	91.98	38.93	8.02	46.73	**9.77**
	Montane	52.59		61.07		53.27	
East Asia	Upland	47.79	77.05	36.92	22.95	45.30	**29.23**
	Montane	52.21		63.08		54.70	
Southeast Asia	Upland	88.23	92.71	84.84	7.29	87.98	**13.14**
	Montane	11.77		15.16		12.02	
West Asia	Upland	20.46	77.75	28.20	22.25	22.18	**32.31**
	Montane	79.54		71.80		77.82	

Supplementary Table 4. Statistics of ecological land loss in highlands induced by human activity expansion.

2000-2020	Highland types	Proportion	The proportion of highland cultivated land expansion induced ecological land loss	Proportion	The proportion of highland artificial surface expansion induced ecological land loss	Proportion	Highland human activity expansion induced ecological land rate
**Asia**	Upland	50.22	90.68	41.87	9.32	**49.46**	**76.09**
	Montane	49.78		58.13		**50.54**	
Central Asia	Upland	37.13	93.92	45.88	6.08	37.66	**75.51**
	Montane	62.87		54.12		62.34	
South Asia	Upland	51.23	97.39	54.07	2.61	51.30	**77.31**
	Montane	48.77		45.93		48.70	
East Asia	Upland	49.12	89.56	41.41	10.44	48.32	**77.15**
	Montane	50.88		58.59		51.68	
Southeast Asia	Upland	88.38	95.71	85.63	4.29	88.26	**94.87**
	Montane	11.62		14.37		11.74	
West Asia	Upland	21.82	88.38	29.90	11.62	22.76	**62.45**
	Montane	78.18		70.10		77.24	

Supplementary Table 5. Contribution rate of cultivated land net growth in highlands for conserving total cultivated land. Negative value (-) means contribution rate of cultivated land net growth in highlands for preventing total net loss of cultivated land; positive value (+) means contribution rate of cultivated land net growth in highlands for conserving total net growth of cultivated land; and dotted line (----) means no contribution rate due to no net cultivated land growth in highlands (the unit of cultivated land area: km^2).

2000-2020	Landforms	2000	2020	Net change (2000-2020)	Highland net growth (upland+ montane)	Total net change （lowland+ highland）	Highland contribution rate
	Lowland	5955741.21	5899720.48	-56020.73			
**Asia**	Upland	415837.77	421885.22	6047.45	**19631.20**	-36389.53	**-53.95**
	Montane	614227.51	627811.26	13583.75			
	Lowland	543994.09	552318.87	8324.78			
Central Asia	Upland	9074.37	9183.34	108.97	244.21	8568.98	**+2.85**
	Montane	14582.60	14717.84	135.23			
	Lowland	2294303.88	2289283.43	-5020.45			
South Asia	Upland	37694.48	39345.48	1650.99	3848.15	-1172.30	**-328.26**
	Montane	48054.58	50251.73	2197.15			
	Lowland	1621069.72	1551350.62	-69719.10			
East Asia	Upland	255383.20	252916.17	-2467.02	-34.81	-69753.90	**----**
	Montane	324445.44	326877.65	2432.21			
	Lowland	910267.00	923402.61	13135.62			
Southeast Asia	Upland	51945.16	58929.59	6984.43	7482.72	20618.33	**+36.29**
	Montane	12046.75	12545.04	498.29			
	Lowland	586106.53	583364.94	-2741.58			
West Asia	Upland	61740.56	61510.64	-229.92	8090.94	5349.36	**+151.25**
	Montane	215098.14	223419.01	8320.86			

Supplementary Table 6. Contribution rate of cultivated land net growth in highlands for conserving total cultivated land at country level. Negative value (-) means contribution rate of cultivated land net growth in highlands for preventing total net loss of cultivated land; positive value (+) means contribution rate of cultivated land net growth in highlands for conserving total net growth of cultivated land; and dotted line (----) means no contribution rate due to no net cultivated land growth in highlands (the unit of cultivated land area: km^2).

Countries	Landforms	2000	2020	Net change (2000-2020)	Highland net growth (upland+ montane)	Total net change (lowland+ highland)	Highland contribution rate
	Lowland	423498.81	431464.60	7965.80			
Kazakhstan	Upland	6595.19	6779.24	184.05	365.23	8331.03	**+4.38%**
	Montane	3189.64	3370.82	181.18			
	Lowland	8571.51	8491.97	-79.54			
Kyrgyzstan	Upland	1074.41	985.88	-88.53	-303.70	-383.25	----
	Montane	8451.98	8236.80	-215.18			
	Lowland	8827.44	8872.65	45.21			
Tajikistan	Upland	528.96	533.52	4.56	146.79	192.00	**+76.45%**
	Montane	2038.42	2180.65	142.23			
	Lowland	33971.63	36087.43	2115.80			
Turkmenistan	Upland	66.60	66.85	0.25	2.98	2118.78	**+0.14%**
	Montane	15.81	18.53	2.73			
	Lowland	69124.71	67402.22	-1722.48			
Uzbekistan	Upland	809.21	817.85	8.64	32.91	-1689.57	**-1.95%**
	Montane	886.76	911.04	24.27			
	Lowland	91429.43	84759.20	-6670.22			
Bangladesh	Upland	177.73	171.03	-6.70	-6.70	-6676.92	----
	Montane	0.00	0.00	0.00			
	Lowland	176.82	196.73	19.91			
Bhutan	Upland	202.67	294.79	92.11	223.93	243.84	**+91.83%**
	Montane	622.47	754.29	131.82			
	Lowland	1912951.21	1910977.96	-1973.24			
India	Upland	24763.63	25608.28	844.65	1103.93	-869.31	**-126.99%**
	Montane	19723.58	19982.87	259.29			
	Lowland	253489.67	254861.16	1371.50			
Pakistan	Upland	4782.10	4944.75	162.65	1057.73	2429.23	**+43.54%**
	Montane	13802.41	14697.48	895.07			
	Lowland	0.00	0.00	0.00			
Maldives	Upland	0.00	0.00	0.00	0.00	0.00	----
	Montane	0.00	0.00	0.00			
	Lowland	19946.53	19730.87	-215.66			
Nepal	Upland	6918.11	7524.97	606.85	**1530.27**	1314.62	**116.4%**
	Montane	13692.29	14615.71	923.42			
	Lowland	16310.59	18757.86	2447.27			
Sri Lanka	Upland	850.25	801.67	-48.58	-61.02	2386.25	----
	Montane	213.82	201.38	-12.44			
	Lowland	1490256.52	1433368.18	-56888.34			
China	Upland	234865.56	237531.31	2665.76	**5029.31**	-51859.03	**-9.7%**
	Montane	320304.71	322668.26	2363.55			
	Lowland	66719.33	64220.42	-2498.92			
Japan	Upland	6089.54	5991.79	-97.75	-36.66	-2535.58	----
	Montane	133.22	194.30	61.09			
	Lowland	29606.45	24661.93	-4944.52			
South Korea	Upland	3951.09	2776.02	-1175.07	-1175.04	-6119.56	----
	Montane	0.27	0.29	0.03			
	Lowland	7906.09	9037.34	1131.25			

Mongolia	Upland	2387.72	2671.05	283.33	452.09	1583.34	**+28.55%**
	Montane	3296.44	3465.19	168.75			
	Lowland	26581.33	20062.76	-6518.57			
North Korea	Upland	8089.29	3946.00	-4143.29	-4304.50	-10823.07	----
	Montane	710.81	549.60	-161.21			
	Lowland	35.97	41.20	5.23			
Brunei	Upland	2.14	2.84	0.69	0.69	5.92	**+11.71%**
	Montane	0.00	0.00	0.00			
	Lowland	57472.32	70285.11	12812.79			
Cambodia	Upland	484.03	963.96	479.93	479.94	13292.72	**+3.61%**
	Montane	0.03	0.04	0.00			
	Lowland	356.03	499.06	143.04			
East Timor	Upland	16.61	16.30	-0.31	-0.33	142.71	----
	Montane	0.02	0.00	-0.02			
	Lowland	17782.28	21330.31	3548.03			
Laos	Upland	1441.14	2173.56	732.41	796.67	4344.70	**+18.34%**
	Montane	264.06	328.32	64.26			
	Lowland	225627.34	221234.65	-4392.69			
Indonesia	Upland	22397.02	22196.72	-200.30	-254.06	-4646.75	----
	Montane	6012.42	5958.67	-53.75			
	Lowland	21718.60	23431.46	1712.86	-123.92	1588.94	----
Malaysia	Upland	1973.07	1847.53	-125.54			
	Montane	58.57	60.18	1.62			
	Lowland	153181.94	153311.70	129.75			
Myanmar	Upland	7771.24	8149.18	377.94	394.01	523.76	**+75.23%**
	Montane	4557.40	4573.46	16.06			
	Lowland	59622.82	62206.49	2583.67			
Philippines	Upland	4994.16	5272.53	278.37	260.59	2844.26	**+9.16%**
	Montane	532.04	514.26	-17.78			
	Lowland	111.16	99.40	-11.77			
Singapore	Upland	6.67	5.39	-1.28	-1.28	-13.04	----
	Montane	0.00	0.00	0.00			
	Lowland	257126.71	252188.86	-4937.85			
Thailand	Upland	4788.25	5795.96	1007.71	1024.79	-3913.06	**-26.19%**
	Montane	127.62	144.70	17.08			
	Lowland	117231.90	118774.46	1542.57			
Vietnam	Upland	8070.82	12505.62	4434.79	**4905.61**	6448.18	**+76.08%**
	Montane	494.59	965.41	470.82			
	Lowland	31245.84	31820.23	574.38			
Afghanistan	Upland	4202.81	4459.89	257.08	**2082.16**	2656.55	**+78.38%**
	Montane	23120.62	24945.71	1825.09			
	Lowland	1798.08	1773.94	-24.15			
Armenia	Upland	516.64	522.16	5.52	163.17	139.02	**+117.37%**
	Montane	4765.72	4923.36	157.65			
	Lowland	28279.27	28396.80	117.53			
Azerbaijan	Upland	1116.17	1115.62	-0.55	-39.25	78.28	----
	Montane	849.54	810.84	-38.70			
	Lowland	45.77	39.03	-6.74			
Bahrain	Upland	0.00	0.00	0.00	0.00	-6.74	----
	Montane	0.00	0.00	0.00			
	Lowland	4588.16	4363.54	-224.63			
Cyprus	Upland	202.60	204.05	1.45	10.74	-213.89	**-5.02%**
	Montane	0.00	9.29	9.29			
	Lowland	14502.42	13646.94	-855.48			
Georgia	Upland	1700.63	1448.18	-252.45	-414.50	-1269.97	----
	Montane	2627.33	2465.28	-162.05			
	Lowland	89673.84	90127.81	453.97			
Iran	Upland	18499.26	18950.23	450.97	**4001.16**	4455.14	**+89.81%**
	Montane	122000.48	125550.67	3550.19			
	Lowland	94348.72	99857.57	5508.85			
Iraq	Upland	505.35	533.00	27.65	30.27	5539.12	**+0.55%**
	Montane	188.35	190.97	2.62			
	Lowland	4947.64	4618.80	-328.84			

Israel	Upland	134.97	107.23	-27.74	-28.76	-357.60	----
	Montane	1.31	0.29	-1.02			
	Lowland	4234.77	4046.09	-188.68			
Jordan	Upland	321.04	259.07	-61.97	-165.21	-353.89	----
	Montane	165.98	62.74	-103.24			
	Lowland	419.15	505.31	86.16			
Kuwait	Upland	0.49	1.31	0.82	0.82	86.98	**+0.95%**
	Montane	0.00	0.00	0.00			
	Lowland	2355.49	1680.24	-675.25			
Lebanon	Upland	431.45	260.49	-170.96	-117.71	-792.96	----
	Montane	192.74	245.99	53.25			
	Lowland	1422.91	1599.13	176.22			
Oman	Upland	3.63	9.25	5.62	5.62	181.84	**+3.09%**
	Montane	0.47	0.47	0.00			
	Lowland	1419.73	843.13	-576.60			
Palestine	Upland	77.20	19.72	-57.48	-57.48	-634.08	----
	Montane	0.00	0.00	0.00			
	Lowland	133.61	196.03	62.42			
Qatar	Upland	0.01	0.03	0.03	0.03	62.45	**+0.04%**
	Montane	0.00	0.00	0.00			
	Lowland	26894.31	26937.36	43.05			
Saudi Arabia	Upland	344.49	381.00	36.51	80.34	123.39	**+65.11%**
	Montane	1147.19	1191.02	43.83			
	Lowland	62782.94	59850.58	-2932.36			
Syria	Upland	1356.11	1352.73	-3.38	166.24	-2766.11	**-6.01%**
	Montane	849.55	1019.18	169.63			
	Lowland	205168.55	199842.34	-5326.21			
Turkey	Upland	31894.50	31164.83	-729.68	498.57	-4827.64	**-10.33%**
	Montane	55426.44	56654.69	1228.25			
	Lowland	1859.30	2235.58	376.28			
United Arab Emirates	Upland	8.55	11.19	2.64	2.64	378.91	**+0.70%**
	Montane	0.00	0.00	0.00			
	Lowland	9986.06	10984.54	998.48			
Yemen	Upland	424.65	710.66	286.01	**1872.09**	2870.56	**+65.22%**
	Montane	3762.44	5348.52	1586.08			

Supplementary Table 7. Population capacity of artificial surface expansion in 2000-2020 (unit: per person).

Study areas	Lowland	Upland	Montane	Total	Highland population capacity rate
**Asia**	409027895	**15553633**	**23997812**	448579342	**8.82%**
Central Asia	3809891	73367	137601	4020859	**5.25%**
South Asia	92038134	1290078	1945652	95273865	**20.54%**
East Asia	207560609	9992776	14472920	232026305	**3.40 %**
Southeast Asia	70152739	1761335	712124	72626198	**10.54%**
West Asia	35466522	2436077	6729515	44632115	**3.41 %**

Supplementary Table 8. Population capacity of artificial surface expansion at country level in 2000-2020 (unit: per person).

Countries	Lowland	Upland	Montane	Total	Highland population capacity rate
Kazakhstan	1368821	23874	5209	1397904	**2.08%**
Kyrgyzstan	233484	29494	93986	356964	**34.59%**
Tajikistan	278302	9684	30149	318135	**12.52%**
Turkmenistan	366109	580	12	366701	**0.16%**
Uzbekistan	1563175	9736	8245	1581156	**1.14%**
Bangladesh	14652545	22878	0	14675423	**0.16%**
Bhutan	7919	740	12369	21028	**62.34%**
India	60500641	625664	495472	61621777	**1.82%**
Pakistan	14097496	218230	479366	14795092	**4.72%**
Maldives	16959	158	0	17117	**0.92%**
Nepal	972439	384519	933711	2290669	**57.55%**
Sri Lanka	1790168	37890	24734	1852792	**3.38%**
China	197394692	9427940	14231092	221053724	**10.7%**
Japan	4246284	171475	184	4417943	**3.89%**
South Korea	4447257	240704	0	4687961	**5.13%**
Mongolia	80224	59983	229118	369325	**78.28%**
North Korea	1392153	92675	12526	1497354	**7.03%**
Brunei	16976	1136	0	18112	**6.27%**
Cambodia	1373255	15384	0	1388639	**1.11%**
East Timor	194751	22612	0	217363	**10.4%**
Indonesia	28753004	711151	439756	29903911	**3.85%**
Laos	711835	35921	6194	753950	**5.59%**
Malaysia	3736048	321885	515	4058448	**7.94%**
Myanmar	6804262	160204	190749	7155215	**4.9%**
Philippines	5381448	104507	51115	5537070	**2.81%**
Singapore	357805	28103	0	385908	**7.28%**
Thailand	11468482	96384	12239	11577105	**0.94%**
Vietnam	11354884	264049	11556	11630489	**2.37%**
Afghanistan	1687722	80285	830989	2598996	**35.06%**
Armenia	125460	31613	88806	245879	**48.97%**
Azerbaijan	877506	23696	30543	931745	**5.82%**
Bahrain	59839	23	0	59862	**0.04%**
Cyprus	102200	512	3	102715	**0.50%**
Georgia	230465	12155	12527	255147	**9.67%**
Iran	5051407	1080982	3900289	10032678	**49.65%**
Iraq	6292440	53747	12029	6358216	**1.03%**
Israel	786714	40043	141	826898	**4.86%**
Jordan	1070064	70715	20646	1161425	**7.87%**
Kuwait	375313	439	0	375752	**0.12%**
Lebanon	910869	223024	49023	1182916	**23.00%**
Oman	716637	5307	636	722580	**0.82%**
Palestine	480934	49141	0	530075	**9.27%**
Qatar	373066	71	0	373137	**0.02%**
Saudi Arabia	3380704	151452	390493	3922649	**13.82%**
Syria	2058868	49692	42213	2150773	**4.27%**
Turkey	7573951	520145	780962	8875058	**14.66%**
United Arab Emirates	2601248	4561	0	2605809	**0.18%**
Yemen	711114	38477	570215	1319806	**46.12%**

Supplementary Table 9. Dominant and potential drivers of human activity expansions in the highlands in different Asian countries during 2000 to 2020.

<table>
<tr><th>Degrees of human activity expansion</th><th>Countries</th><th colspan="3">Dominant and potential drivers</th></tr>
<tr><td>Extremely high (≈90%) or very low human activity expansion rates in the highlands (<2%)</td><td>Bhutan, Qatar, Bahrain, Bangladesh, Maldives, Iraq, Turkmenistan, Kuwait, United Arab Emirates</td><td colspan="3">In Bhutan, flat land resources are scarce and more than 95% of its land is highlands (Fig.2d). Thus, highland development is obligatory for maintaining social and economic developments. Such situation explains the extremely high human activity expansion rate in the highlands in Bhutan (≈90%). In contrast, the scarce highlands in some countries (highland proportion <5% in Qatar, Bahrain, Bangladesh, Maldives, Iraq, Turkmenistan, Kuwait and United Arab Emirates, Supplementary Fig.12a) can explain their low human activity expansion rates in the highlands (<2%). Therefore, topographic factors significantly impact highland developments in the abovementioned countries (R^2 = 0.9981, $p < 0.01$, Supplementary Fig 12b).</td></tr>
<tr><td>Very high human activity expansion rate in the highlands (~38% -76%)</td><td>Nepal, Armenia, Iran, Yemen, Afghanistan, Kyrgyzstan, Mongolia, Turkey, North Korea</td><td colspan="2">Nepal, Armenia, Iran, Afghanistan, Kyrgyzstan, Mongolia, Turkey and North Korea have high highland rates (proportion of highlands > 57%, Fig. 2d), therefore the human activity expansions in the highlands are natural phenomenon. Since 2006, a nationwide “Development of Pastures and Meadows and Pasture and Forage Crop Production Project” in Turkey contributed to the agricultural developments in the highlands[1]. Mongolia’s western, northern and northeastern parts are upland and suitable for farming, and a national project for the development of farming was started since 1997 to improve food security and increase family incomes to alleviate poverty, which has largely driven Mongolia's highland developments[2]. Notably, Yemen has the least highland areas (only accounts for 40.35%) among these countries, while the highland developments was > 55% (Fig. 2d), which is mainly due to following reasons: most of the lowlands (e.g. coastal plains) are semi-desert, and the highlands are fertile and suitable for developments; and Yemen’s civil war exacerbated long-term political instability and economic backwardness, thus Yemen strengthened the developments and utilizations of highlands (including agricultural developments, oil /gas /mineral explorations) to promote livelihoods and economic recovery[3-5].</td><td rowspan="2">Highlands are rich in agro-biodiversity and genetic resources, and Central and West Asia countries (i.e. Afghanistan, Iran, Tajikistan, Turkey, Pakistan, Azerbaijan and Georgia,) have built collaboration on rainfed agricultures in highlands with the International Center for Agricultural Research in the Dry Areas to support growing populations and reduce poverty[1].

The transitional economy for Central Asian countries and North Korea (i.e. transformation from planned economy to market economy or mixed economy) have boosted the developments of infrastructure, industry and agriculture, while strengthening the utilizations of resources and export trade[6], which may lead to the widespread highland developments of Kyrgyzstan, Tajikistan, Pakistan, Uzbekistan Kazakhstan and North Korea.

From 2009, Armenia, Azerbaijan and Georgia participated in the part of the World Bank’s Europe and Central Asia (ECA) Regional Analytical and Advisory Activities Program on Reducing Vulnerability to Climate Change in ECA Agricultural Systems program, of which more systematic land management (i.e. shifting crops from lowlands to highlands, away from areas vulnerable to</td></tr>
<tr><td>High human activity expansion rate in the highlands (>Asia level) (~23% -34%)</td><td>Lebanon, Tajikistan, China, Vietnam, Georgia</td><td>Lebanon faces water stress and food insecurity, while its highlands have abundant rainfall (~1400 mm average annual rainfall), which drive Lebanon to develop highlands to a certain extent[15].The ‘Development of the Western Region’ policy (comprising 15 mountainous provinces and regions in the central and western China) issued in 2000 and the ‘Low-slope Hilly Regions Comprehensive</td><td>Marketing demands (e.g. timber export, expansions of cash crops-corn, tea, coffee and upland rice), land scarcity (by 2000, most of the mainland Southeast</td></tr>
</table>

<table>
<tr>
<td></td>
<td></td>
<td>Development and Utilization' policy formulated in 2006 considerably contributed to the human activity expansion rate in the highlands in China[9,16].</td>
<td rowspan="3">Asian's lowlands were used for some form of agriculture) and national land-tenure policies intensified the expansions of cultivated lands and artificial surfaces in the highlands of Southeast Asian countries (e.g. Vietnam, Malaysia, Indonesia, Philippines, Thailand, Laos, East Timor, Brunei and Cambodia)[17,18-21].</td>
<td rowspan="3">drought and flooding from sea level rise) drove highland developments in these countries[7].

Since the 21st century, the global urbanization has been accelerating significantly (urban population has increased from 30% in 1950 to 56% in 2019), especially in Asia and Africa[8]. To meet the needs of urban population growth and rapid urbanization as well as people's pursuit of fresh air, landscape, exclusivity and closeness to nature, the hillsides around urban regions are being developed at an accelerating rate in different Asian cities[9,10,11,12], which drove the highland developments in many Asian countries.

China's "One Belt One Road" initiative increased direct investments and the construction of various infrastructures in Asian countries (e.g. railways, highways, hydropower stations, natural gas pipeline projects), which contributed to the highland developments in Asian countries to a certain extent[13].

Highlands have beautiful natural landscapes, and Asian countries have accelerated the developments of mountain tourisms in the 21st century, which also drove highland developments to a certain extent[14].</td>
</tr>
<tr>
<td>Medium human activity expansion rate in the highlands (<Asia level) (~5% -20%)</td>
<td>Pakistan, Laos, Cyprus, Japan, South Korea, Myanmar, Philippines, Palestine, Indonesia, Jordan, Thailand, Saudi Arabia, Malaysia, Syria, Azerbaijan, Brunei, India, Uzbekistan, East Timor</td>
<td>In order to maintain/protect mountain agriculture, Japan promulgated the Depopulated Areas Emergency Act and the Mountain Villages Development Act, which promoted the developments of highlands in Japan[2].Highland farming was expanded to produce commercial crops and has become a major income source for farm households, which can drive highland developments in South Korea[2]. Since 2004, the Annan peace plan led to a construction boom in Cyprus, and then induced disorderly land developments[22]. After joining the World Trade Organization in 1999, Jordan strengthened economic regulation and control, and took corresponding measures in finance, infrastructure, investment attraction and foreign aid[23], which may drive the developments of highlands. India adopted watershed approach based on the principle of people's participation to develop the uplands in Indian Himalayas and south India[2].</td>
</tr>
<tr>
<td>Low human activity expansion rate in the highlands (<Asia level) (~2% -5%)</td>
<td>Israel, Singapore, Cambodia, Sri Lanka, Kazakhstan</td>
<td>These countries have relatively scarce highlands (Fig. 2d), therefore most human activities are concentrated in lowlands.</td>
</tr>
</table>

Supplementary Table 10. Criteria used to determine major landforms based on broad physiographic features (sources, summarized by Margono et al.[24]).

Categories	Major landforms	Broad physiographic features	Criteria of elevation and slope classes
Lowland	Lowland	Plains and terraces	elevation 0-1000m with slope 0-15°
			elevation 1000-1200m with slope 0-8°
Highland	Upland	Hills	elevation 0-1000 m with slope >15°
			elevation 1000-1200 m with slope >8°
			elevation 1200-1500 with slope 0-3°
	Montane	Mountains	elevation 1200-1500 with slope >3°
			and elevation >1500m

Note: a few regions with an elevation of less than 0m in Asia are divided into lowlands in our study.

Supplementary references

1. Roozitalab, M. H., Serghini, H., Keshavarz, A., Eser, V., & Depauw, E. Sustainable Agricultural Development of Highlands in Central, West Asia, and North Africa. Synthesis of Regional Expert Meetng on Highland Agriculture November 2011, Karaj, Iran (2013).
2. Partap, T., & Chancellor, V. Sustainable farming systems in upland areas. Report of the APO study meeting on sustainable farming systems in upland areas, New Delhi, 15–19 Jan 2001. Asian Productivity Organization, Tokyo (2004).
3. Encyclopedia of the Nations. Yemen country profile. Available at: https://www.nationsencyclopedia.com/Asia-and-Oceania/Yemen.html (2022)
4. Yadav, S.P. & Lynch, M. Politics, Governance and Reconstruction in Yemen. (Washington, Pomes Studies, 2018).
5. Sowers, J. & Weinthal, E. Humanitarian challenges and the targeting of civilian infrastructure in the Yemen war. *Int. Aff.* **97**, 157-177 (2021).
6. Park, J., Kang, B., Min, J., Gwun, K.&Yun, C. Economic Development Strategies of Major Central Asian Countries and Their Implications for Korea. KIEP Research Paper, World Economy Brief. Available at: https://ssrn.com/abstract=3089482 (2017).
7. Sutton, W. R., Srivastava, J. P., Neumann, J.E., Strzępek, K. M.& Droogers P. Reducing the vulnerability of Azerbaijan's agricultural systems to climate change: impact assessment and adaptation options (World Bank Publications, Washington, 2013).
8. World bank. World bank database. Available at: https://data.worldbank.org.cn/ (2020).
9. Yang, C. *et al*.. Comparing hillside urbanizations of Beijing-Tianjin-Hebei, Yangtze River Delta and Guangdong–Hong Kong–Macau greater Bay area urban agglomerations in China. *Int. J. Appl. Earth Obs. Geoinfor.* **102**, 102460 (2021).
10. Yang, C. *et al*. Characteristics and trends of hillside urbanization in China from 2007 to 2017. *Habitat Int.* **120**, 102502 (2022).
11. Too, E. G., Adnan, N. & Trigunarsyah, B. Project governance in Malaysia hillside developments. 6th International Conference on Construction in the 21st Century (CITC-VI), Kuala Lumpur Malaysia. (2011).
12. Ahn, J. E. Cities on the Edge: Signifcance and Preservation of Hillside Squatter Settlements in Korea (Columbia University, New York, 2014).
13. Foo, N., Lean, H. H. & Salim, R. The impact of China's One Belt One Road initiative on international trade in the ASEAN region. *N. Am. J. Econ. Financ.* **54**,101089 (2019).
14. Bui, H. T., Jones, T.E. & Apollo, M. Nature-Based Tourism in Asia’s Mountainous Protected Areas

(Geographies of Tourism and Global Change, Springer, Cham, 2021).

15. Kallas, G., Palacios-Rodriguez, G. & Kattar, S. Land Suitability for Biological Wastewater Treatment in Lebanon and the Litani River Basin Using Fuzzy Logic and Analytical Hierarchy Process. *Forests* **13**, 139 (2022).
16. Lu, S., Song, X, Wang, B.Y. Development of the Western Region. Available at https://m.gmw.cn/baijia/ 2021-04/06/34741100.html (2021).
17. Zeng, Z. Z. *et al.* Highland cropland expansion and forest loss in Southeast Asia in the twenty-first century. *Nat. Geosci.* **11**, 556-565 (2018).
18. Bruun, T. B., Neergaard, A., Lawrence, D. & Ziegler, A. D. Environmental Consequences of the Demise in Swidden Cultivation in Southeast Asia: Carbon Storage and Soil Quality. *Hum. Ecol.* **37**, 375-388 (2009).
19. Bruun, T. B., *et al*. Intensifcation of upland agriculture in Thailand: development or degradation? . *Land Degrad. Dev.* **28**, 83–94 (2017).
20. Schmidt-Vogt, D. *et al.* An Assessment of Trends in the Extent of Swidden in Southeast Asia. *Hum. Ecol.* **37**, 269-280 (2009).
21. Byerlee, D., Stevenson, J. & Villoria, N. Does intensification slow crop land expansion or encourage deforestation? *Glob. Food Secur.* **3**, 92-98 (2014).
22. Yorucu, V., & Keles, R. The construction boom and environmental protection in northern Cyprus as a consequence of the Annan plan . *Constr. Manag. Econ.* **25**, 77-86 (2007).
23. MOFA. The Hashemite Kingdom of Jordan. Available at: http://www.fmprc.gov.cn/ (2021).
24. Margono, B. A., Potapov, P. V., Turubanova, S., Stolle, F. & Hansen, M. C. Primary forest cover loss in Indonesia over 2000-2012. *Nat. Clim. Change* **4**, 730-735 (2014).
