# Supplementary material for: Human expansion into Asian highlands in the 21st Century and its effects
Source: Nat Commun. 2022 Aug 24;13:4955. doi: 10.1038/s41467-022-32648-8 (PMC9402921; doi:10.1038/s41467-022-32648-8)
Supplement: Supplementary file 3 — Description of Additional Supplementary Files [file 41467_2022_32648_MOESM3_ESM.pdf]

Description of Additional Supplementary Files

File Name: Supplementary Data 1
Description: The datatset of population, GDP and economic levels in 48 Asian countries.

File Name: Supplementary Data 2
Description: Visual interpretation of high resolution satellite imageries at 6676 sample sites randomly selected from human activity expansion areas in highlands in Asia.

File Name: Supplementary Data 3
Description: Visual interpretation of high resolution satellite imageries at 4008 sample sites randomly selected from human activity expansion areas in lowlands in Asia.

File Name: Supplementary Data 4
Description: Visual interpretation of high resolution satellite imageries at 6275 sites randomly selected from human activity expansion induced-ecological land loss areas in highlands across Asia.

File Name: Supplementary Data 5
Description: Visual interpretation of high resolution satellite imageries at 4474 sites randomly selected from human activity expansion induced-ecological land loss areas in lowlands across Asia.
